# Excess TGF-β1 Drives Cardiac Mesenchymal Stromal Cells to a Pro-Fibrotic Commitment in Arrhythmogenic Cardiomyopathy

**DOI:** 10.3390/ijms22052673

**Published:** 2021-03-06

**Authors:** Angela Serena Maione, Ilaria Stadiotti, Chiara Assunta Pilato, Gianluca Lorenzo Perrucci, Valentina Saverio, Valentina Catto, Giulia Vettor, Michela Casella, Anna Guarino, Gianluca Polvani, Giulio Pompilio, Elena Sommariva

**Affiliations:** 1 Vascular Biology and Regenerative Medicine Unit, Centro Cardiologico Monzino IRCCS, 20138 Milan, Italy; ilaria.stadiotti@ccfm.it (I.S.); chiarapilato91@gmail.com (C.A.P.); gianluca.perrucci@ccfm.it (G.L.P.); valentina.saverio@uniupo.it (V.S.); giulio.pompilio@ccfm.it (G.P.); elena.sommariva@ccfm.it (E.S.); 2 Cardiac Arrhythmia Research Centre, Centro Cardiologico Monzino IRCCS, 20138 Milan, Italy; valentina.catto@ccfm.it (V.C.); giulia.vettor@ccfm.it (G.V.); michelacasella@hotmail.com (M.C.); 3 Cardiovascular Tissue Bank of Milan, Centro Cardiologico Monzino IRCCS, 20138 Milan, Italy; anna.guarino@ccfm.it (A.G.); gianluca.polvani@ccfm.it (G.P.); 4 Department of Biomedical, Surgical and Dental Sciences, Università degli Studi di Milano, 20122 Milan, Italy

**Keywords:** arrhythmogenic cardiomyopathy, cardiac-mesenchymal stromal cells, fibrosis, TGF-β1, cardiac remodeling

## Abstract

Arrhythmogenic Cardiomyopathy (ACM) is characterized by the replacement of the myocardium with fibrotic or fibro-fatty tissue and inflammatory infiltrates in the heart. To date, while ACM adipogenesis is a well-investigated differentiation program, ACM-related fibrosis remains a scientific gap of knowledge. In this study, we analyze the fibrotic process occurring during ACM pathogenesis focusing on the role of cardiac mesenchymal stromal cells (C-MSC) as a source of myofibroblasts. We performed the ex vivo studies on plasma and right ventricular endomyocardial bioptic samples collected from ACM patients and healthy control donors (HC). In vitro studies were performed on C-MSC isolated from endomyocardial biopsies of both groups. Our results revealed that circulating TGF-β1 levels are significantly higher in the ACM cohort than in HC. Accordingly, fibrotic markers are increased in ACM patient-derived cardiac biopsies compared to HC ones. This difference is not evident in isolated C-MSC. Nevertheless, ACM C-MSC are more responsive than HC ones to TGF-β1 treatment, in terms of pro-fibrotic differentiation and higher activation of the SMAD2/3 signaling pathway. These results provide the novel evidence that C-MSC are a source of myofibroblasts and participate in ACM fibrotic remodeling, being highly responsive to ACM-characteristic excess TGF-β1.

## 1. Introduction

Arrhythmogenic Cardiomyopathy (ACM) is a rare genetic cardiac disease [1], mostly inherited with autosomic dominant traits [2] and is mainly due to mutation in genes encoding cardiac desmosomes. ACM hearts are characterized by cardiomyocytes (CM) death, replacement of the myocardium with fibrotic or fibro-fatty tissue, and inflammatory infiltrates. The ventricular fibrotic and fibro-fatty substitution are segmental or irregular among patches of CM [3,4] and generally progress from the epicardium to the endocardium, provoking structural and functional myocardial alterations [5]. The ACM tissue heterogeneity may, in turn, cause re-entrant electrical activity, contributing to ventricular arrhythmias and causing, in the worst-case scenario, sudden cardiac death. The molecular mechanisms of fibrosis are well known for different cardiac diseases but few studies are focused on ACM-specific pro-fibrotic processes [6].

In general, the cardiac fibrosis process is thought to start as a protective mechanism against injury and grows in excessive collagen deposition, leading to myocardial scar formation [4]. Typical events envisage the differentiation of fibroblast into myofibroblast leading to extracellular matrix (ECM) deposition. Different cells can be considered myofibroblast precursors, among which resident cardiac fibroblasts [7,8,9] and cardiac mesenchymal stromal cells (C-MSC) [10,11] can be mentioned. In this context, the typical pro-fibrotic factor transforming growth factor-β (TGF-β) can promote fibrotic remodeling [12,13].

Adipogenesis and fibrogenesis are characterized by two distinct differentiation programs that are finely regulated by independent pathways. The adipogenic process occurring in ACM hearts can be ascribed to C-MSC ability in differentiating into adipocytes [14]. This event is characterized by the enhanced expression of PPARγ that is the master regulator of adipogenic differentiation [15] and acts preventing myofibroblasts differentiation and collagen deposition [16]. On the contrary, TGF-β1 was shown, in lungs, to induce fibroblast to myofibroblast differentiation, reducing in parallel with the expression of PPARγ [17]. Interestingly, the fibro-adipogenic progenitors (FAP), a subpopulation of C-MSC, have been associated with the fibro-fatty substitution in ACM based on their bi-potential ability [18]. These cells are mostly characterized by a fibrous commitment and only a small percentage with fat genes expression [18].

The present study highlights the role of the whole C-MSC population [19] in the pro-fibrotic process of ACM human hearts. Here we provide evidence that C-MSCs are conditioned by the excess environmental TGF-β to display typical pro-fibrotic molecular events. The cells, isolated from human ACM hearts and exposed in vitro to TGF-β1 stimulation, are prone to develop the pro-fibrotic phenotype, through the activation of SMAD2/3 signaling.

## 2. Results

### 2.1. Arrhythmogenic Cardiomyopathy Patient-Derived Tissues Exhibit Higher Fibrosis and TGF-β1 Levels Than Healthy Controls

**Figure 1:**
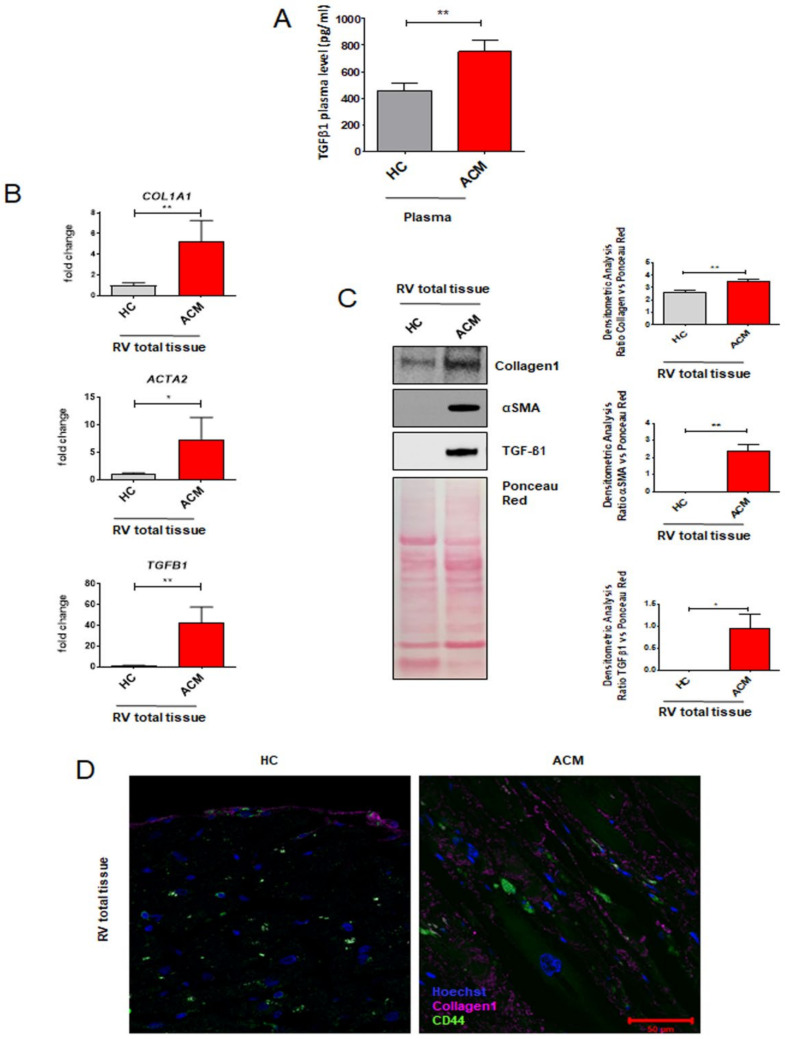
Fibrotic profile characterization of ACM-patient-derived tissues. (**A**) TGF-β1 plasma levels of HC donors and ACM patients. *n* = 52/group. Student’s *t*-test: ** *p* < 0.01. (**B**) Expression of fibrosis-associated genes (*COL1A1, ACTA2, TGFB1*) in total RNA extracts of RV endomyocardial bioptic samples from HC donors and ACM patients. GAPDH was used as a house-keeping gene and qRT-PCR data are presented as the fold change of target gene expression with respect to HC C-MSC. *n* = 4/group. Student’s *t*-test: * *p* < 0.05, ** *p* < 0.01. (**C**) Representative images of Western blot analysis of fibrosis-associated proteins (Collagen1, αSMA, TGF-β1) in total protein extracts of RV endomyocardial bioptic samples from HC donors and ACM patients. Quantification of the protein abundance relative to Ponceau Red is shown in the graphs. *n* = 4/group. Student’s *t*-test: * *p* < 0.05, ** *p* < 0.01. (**D**) Representative images of immunostaining of COL1A1 and CD44 sections from biopsies from HC donors and ACM patients. Nuclei are stained with Hoechst 33342. Magnification is 63X and the scale bar indicates 50 μm. *n* = 3/group.

To characterize the pro-fibrotic profile of ACM patients, we firstly investigated the circulating TGF-β1 levels in plasma collected from ACM patients and healthy control donors (HC). As reported in Figure 1A, ACM patients showed higher circulating levels of TGF-β1 compared with age- and sex-matched HC (Figure 1A: TGF-β1 750.30 ± 84.27 pg/mL in ACM vs. 455.50 ± 55.80 in HC; *n* = 52 each; *p* = 0.0069).

To proceed with the characterization at cardiac tissue level, we collected RV endomyocardial bioptic samples from ACM patients, in which the biopsy procedure was necessary for diagnostic purposes, and control subjects’ right ventricle (RV) postmortem samples. We evaluated the expression of the principal genes involved in fibrosis by using total RNA extracts from RV total tissues. qRT-PCR analyses showed a higher expression in ACM patients’ samples vs. HC of genes *COL1A1*, *ACTA2*, and *TGFB1*, encoding typical pro-fibrotic mediators COL1A1, α-SMA, and TGF-β1, respectively (Figure 1B: *COL1A1* 5.510 ± 0.3082 ΔCt in HC vs. 3.298 ± 0.4727 ΔCt in ACM, *n* = 4 each, *p* = 0.0078; *ACTA2* 8.500 ± 0.2858 ΔCt in HC vs. 6.225 ± 0.8459 ΔCt in ACM, *n* = 4 each, *p* = 0.0436; *TGFB1* 6.900 ± 0.2721 ΔCt in HC vs. 1.998 ± 0.9134 ΔCt in ACM, *n* = 4 each, *p* = 0.0021).

Western blot analysis confirmed that also that COL1A1, α-SMA, and TGF-β1 proteins were expressed at higher level in ACM tissues compared to HC tissues (Figure 1C: COL1A1 2.631 ± 0.1053 a.u. in HC vs. 3.495 ± 0.1680 a.u. in ACM, *n* = 3, *p* = 0.0058; α-SMA 0.001173 ± 7.545 × 10^−5^ a.u. in HC vs. 2.384 ± 0.4025 a.u. in ACM, *n* = 4, *p* = 0.0010; TGF-β1 0.009511 ± 0.005374 a.u. in HC vs. 0.9650 ± 0.3165 a.u. in ACM, *n* = 4, *p* = 0.0235).

To verify the involvement of C-MSC in the fibrotic substitution, we performed an immunofluorescence analysis on ACM and HC RV section by using COL1A1 and CD44 antibodies, as fibrosis and mesenchymal markers, respectively. ACM sections showed a larger collagen deposition area (marked by COL1A1) vs. HC sections and a high number of CD44 positive cells were present in collagen-substituted areas (Figure 1D).

Altogether, these data (i) showed, for the first time, excessive circulating TGF-β1 in ACM patients; (ii) confirmed that ACM hearts show abundant fibrosis, in terms of pro-fibrotic gene and protein expression; and (iii) revealed mesenchymal cell presence in the fibrotic areas.

### 2.2. Cardiac Mesenchymal Stromal Cells Isolated from Arrhythmogenic Cardiomyopathy Patients and from Healthy Controls Exhibit Comparable Fibrotic Marker Levels

Since C-MSC are well described as potential myofibroblast progenitors [10] and immunofluorescence results highlighted the presence of the C-MSC population in the fibrotic region of ACM tissue, we investigated the expression of pro-fibrotic mediators in isolated cells.

**Figure 2:**
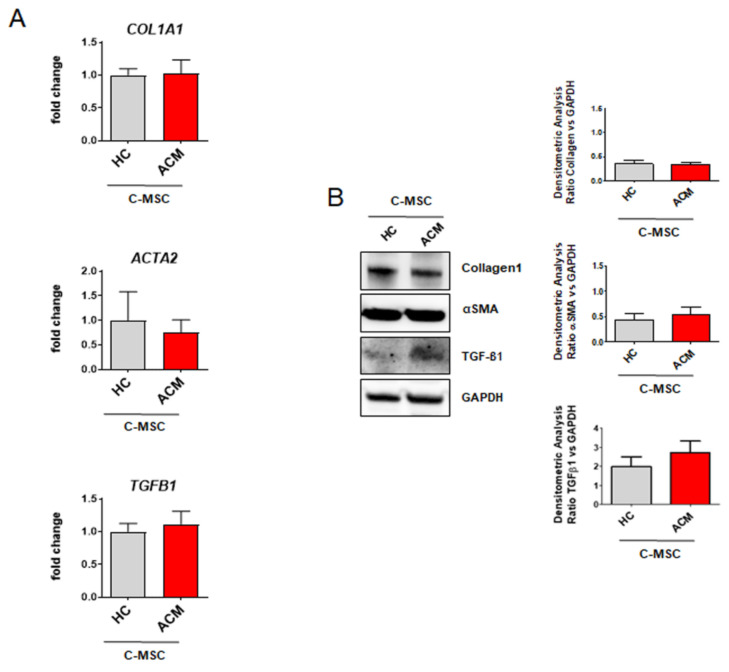
Analysis of fibrotic markers in ACM C-MSC. (**A**) *COL1A1, ACTA2, TGFB1* gene expression in total RNA extracts of cardiac mesenchymal stromal cells isolated from HC donors and ACM patients in culture condition. GAPDH was used as a house-keeping gene and qRT-PCR data are presented as the fold change of target gene expression respect to HC C-MSC. *n* = 7/group. Student’s *t*-test: no significant difference. (**B**) Western blot analysis of Collagen1, αSMA, and TGF-β1 proteins in total extracts of cardiac mesenchymal stromal cells isolated from HC donors and ACM patients in culture condition. Quantification of the protein abundance relative to GAPDH is shown in the graphs. *n* = 4/group. Student’s *t*-test: no significant difference.

C-MSC were isolated from RV endomyocardial bioptic samples from ACM patients and control subjects’ RV samples. As previously shown by gene expression analyses, C-MSC from ACM and HC groups exhibit comparable levels of *COL1A1*, *ACTA2*, and *TGFB1* (Figure 2A). These results were further confirmed by protein expression analysis that showed no significant difference for the collagen1, α-SMA, and TGF-β1 proteins in the two groups (Figure 2B).

These data indicate that ACM C-MSC do not show an evident myofibroblast-like phenotype in growing culture conditions.

### 2.3. TGF-β1 Stimulation Drives Pro-Fibrotic Differentiation of Cardiac Mesenchymal Stromal Cell from Arrhythmogenic Cardiomyopathy Patients

To establish whether the excessive plasma and tissue levels of TGF-β1 could influence C-MSC differentiation into myofibroblasts, we analyzed the fibrotic-like cell phenotype of ACM and HC C-MSC after TGF-β1 in vitro treatment. Besides, to understand whether the cellular response is specifically dependent on TGF-β1 activity, we analyzed the effect of treatment with LY364947 [20], a specific TGF-β1 inhibitor, acting on the TGF-β1 receptor kinase domain [3]. Since we wanted to appreciate the modulation due to the treatments, regardless of the starting values, we graphed gene and protein expression relative to each untreated level.

**Figure 3:**
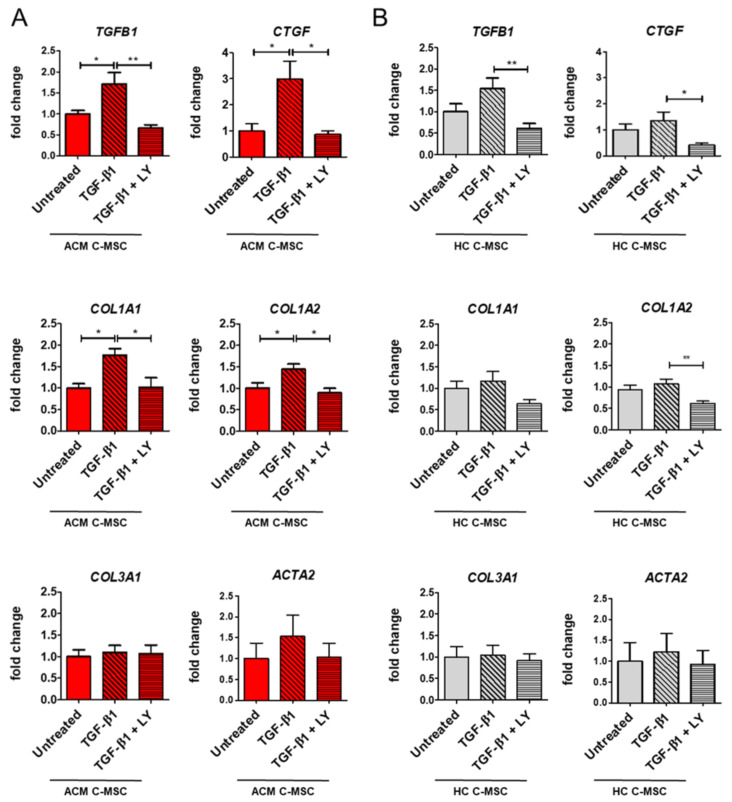
Effect of TGF-β1 stimulation on gene expression of pro-fibrotic markers. C-MSC isolated from HC donors and ACM patients were grown in low serum (2%) overnight and were stimulated or not with TGF-β1 for 24 h in the presence or absence of LY364947 treatment. Comparison of fibrosis-associated gene expression (*TGFB1, CTGF, COL1A1, COL1A2, COL3A1, ACTA2*) in total RNA extracts from ACM (**A**) and HC C-MSC (**B**). GAPDH was used as a house-keeping gene and qRT-PCR data are presented as the fold change of target gene expression with respect to the untreated. *n* = 3/group. One-way ANOVA and Bonferroni’s post-test: * *p* < 0.05, ** *p* < 0.01.

qRT-PCR analyses showed a significant upregulation of *TGFB1*, *CTGF*, *COL1A1*, and *COL1A2* after TGF-β1 stimulation in ACM C-MSC, unlike HC C-MSC (Figure 3A–B). The presence of LY during the TGF-β1 treatment was able to restore the gene expression levels to the basal condition (Figure 3A: *TGFB1* 1.000 ± 0.08193 a.u. in Untreated vs. 1.709 ± 0.2744 a.u. in TGF-β1 vs. 0.6639 ± 0.07966 a.u. in TGF-β1+LY, *n* = 7 each, *p* = 0.0273 and *p* = 0.0013 respectively; *CTGF* 1.000 ± 0.2671 a.u. in Untreated vs. 2.990 ± 0.6841 a.u. in TGF-β1 vs. 0.8638 ± 0.1395 a.u. in TGF-β1 + LY, *n* = 6 each *p* = 0.0158 and *p* = 0.0100 respectively; *COL1A1* 1.000 ± 0.09748 a.u. in Untreated vs. 1.766 ± 0.1504 a.u. in TGF-β1 vs. 1.019 ± 0.2223 a.u. in TGF-β1+LY, *n* = 7 each *p* = 0.0123 and *p* = 0.0147 respectively; *COL1A2* 1.000 ± 0.1305 a.u. in Untreated vs. 1.446 ± 0.1171 a.u. in TGF-β1 vs. 0.8942 ± 0.1067 a.u. in TGF-β1+LY, *n* = 7 each *p* = 0.0479 and *p* = 0.0122 respectively). In ACM C-MSC, the same trend in response to TGF-β1 is evident also for *ACTA1* gene, even if not significant, while we could not detect effects on COL3A1 (Figure 3A).

**Figure 4:**
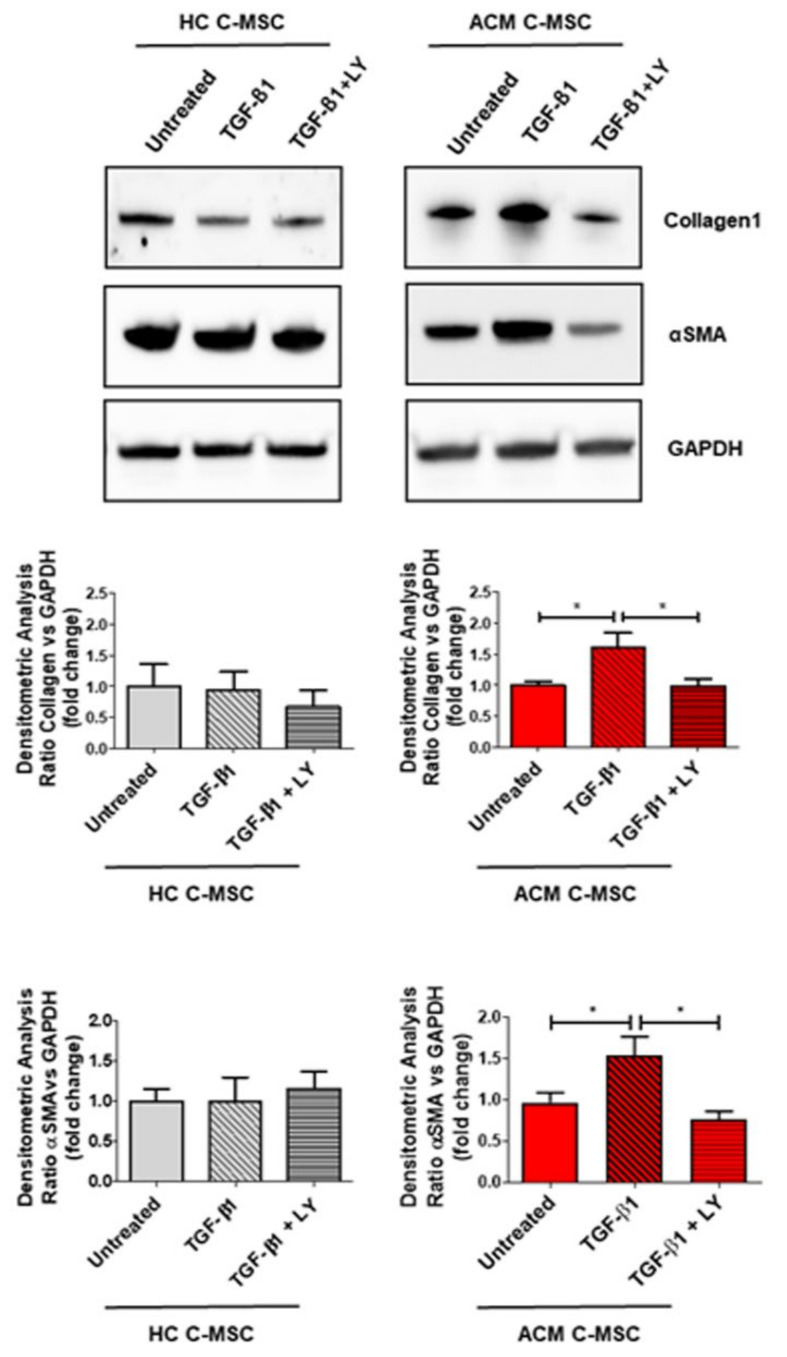
TGF-β1 induces the overexpression of fibrosis-associated proteins. Following overnight low serum (2%) growth, cardiac mesenchymal stromal cells isolated from HC donors and ACM patients were stimulated with TGF-β1 for 5 days in the presence or absence of LY364947 treatment. Representative images of Western blot analysis on fibrosis-associated proteins (Collagen1, αSMA) in total protein extracts of treated cells. Quantification of the protein abundance relative to GAPDH has been shown as fold change respect to untreated protein in the graphs and results are expressed as mean ± SEM, (*n* = 3/group). One-way ANOVA and Bonferroni’s post-test: * *p* < 0.05.

Results on gene expression levels were further confirmed by protein expression analysis, since ACM C-MSC treated with TGF-β1 showed a higher Collagen1 and α-SMA expression levels, which in turn were reverted by adding LY (Figure 4: Collagen1 1.000 ± 0.06145 a.u. in Untreated vs. 1.609 ± 0.2325 a.u. in TGF-β1 vs. 0,9827 ± 0,1274 in TGF-β1 + LY, *n* = 3 each, *p* = 0.0337 and *p* = 0.0304 respectively; α-SMA 1.000 ± 0.1192 a.u. in Untreated vs. 1.534 ± 0.2327 a.u. in TGF-β1 vs. 0.7673 ± 0.09760 a.u. in TGF-β1+LY, *n* = 3 each, *p* = 0.0482 and *p* = 0.0152 respectively).

**Figure 5:**
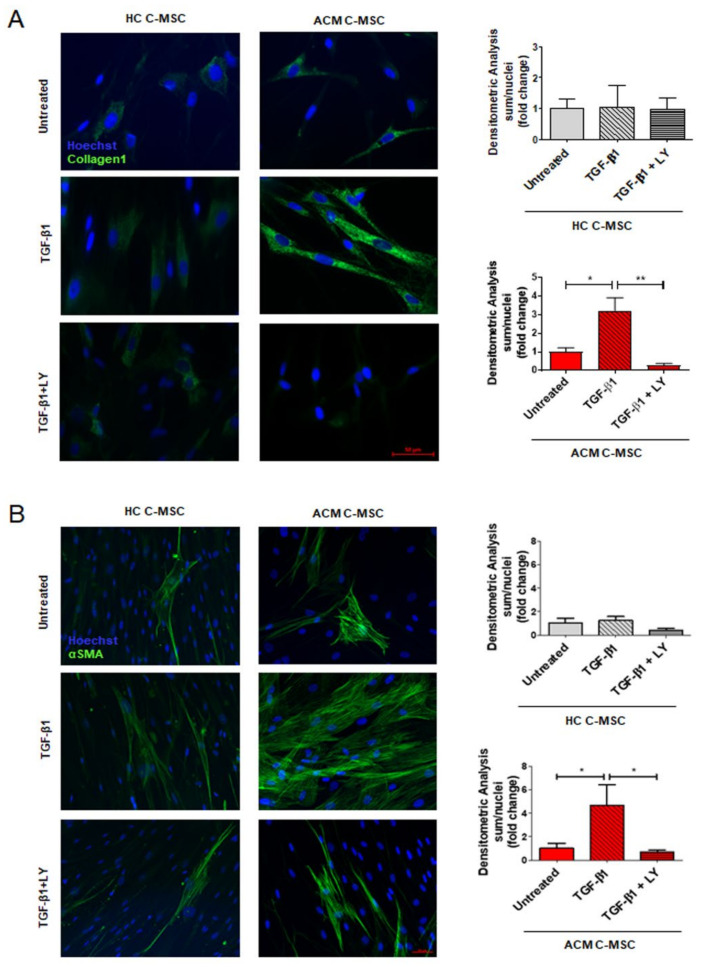
TGF-β1 leads to increased collagen production and α-SMA positive stress fibers in ACM C-MSC. C-MSC isolated from HC donors and ACM patients were treated as described in Figure 4. Representative images of immunostaining for COL1A1 (**A**) and αSMA (**B**). Nuclei are stained with Hoechst 33342. Magnification is 40× and the scale bar indicates 50 μm (*n* = 3/group). Quantification of the images has been shown as fold change respect to untreated in the graphs and results are expressed as mean ± SEM, (*n* = 3/group). One-way ANOVA and Bonferroni’s post-test: * *p* < 0.05, ** *p* < 0.01.

Lastly, the same experimental conditions were applied to analyze collagen deposition and the presence of α-SMA positive stress fibers. Immunofluorescence analysis demonstrated that TGF-β1 can induce differentiation of C-MSC into myofibroblasts by increasing collagen1 (Figure 5A: Collagen1 1.000 ± 0.2215 a.u. in Untreated vs. 3.222 ± 0.7017 a.u. in TGF-β1 vs. 0.2909 ± 0.08525 a.u. in TGF-β1+LY, *n* = 3 each, *p* = 0.0210 and *p* = 0.0014 respectively) and α-SMA production (Figure 5B: α-SMA 1.000 ± 0.4237 a.u. in Untreated vs. 4.683 ± 1.750 a.u. in TGF-β1 vs. 0.7008 ± 0.1695 a.u. in TGF-β1+LY, *n* = 3 each, *p* = 0.0248 and *p* = 0.0165 respectively). Importantly, both these effects occurred exclusively in ACM C-MSC and were abolished by LY treatment.

Overall, these data demonstrate that C-MSC isolated from ACM patients are more responsive to pro-fibrotic stimuli compared to HC C-MSC and are more prone to differentiate into myofibroblasts.

### 2.4. TGF-β1 Treatment Cause Activation of TGF-β1 Canonical Signaling Pathway in Arrhythmogenic Cardiomyopathy-Derived Cardiac Mesenchymal Stromal Cells

**Figure 6:**
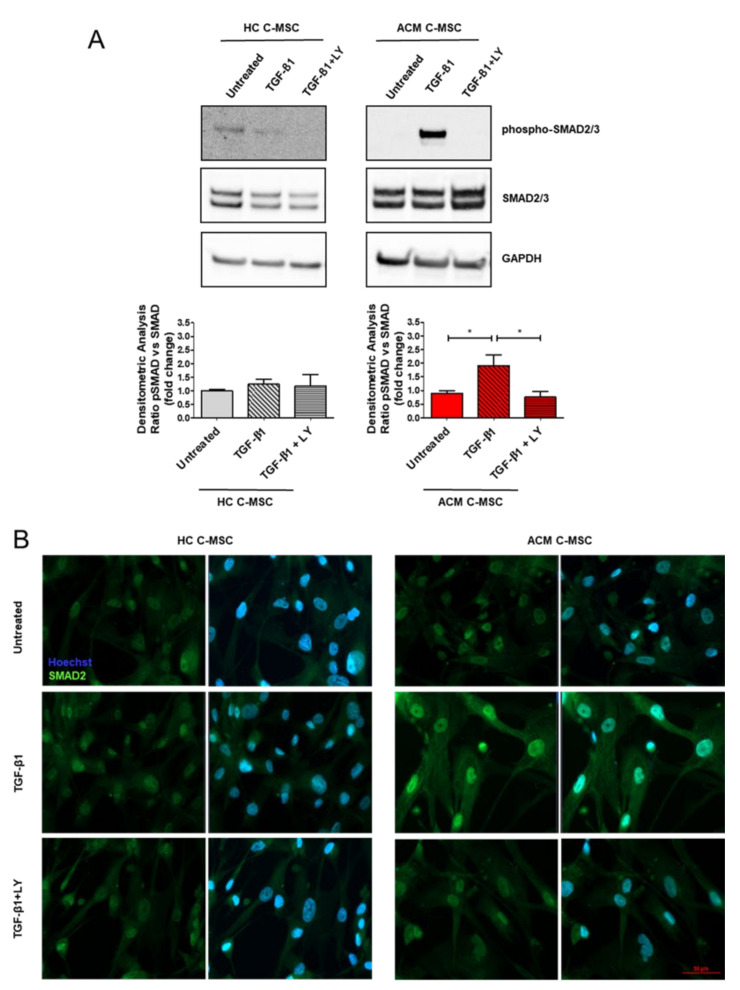
ACM C-MSC pro-fibrotic commitment depends on the TGF-β1 canonical signaling pathway. Cardiac mesenchymal stromal cells isolated from HC donors and ACM patients were grown in low serum (2%) overnight and were stimulated or not with TGF-β1 for 30 min in the presence or absence of LY364947 treatment. (**A**) Representative images of Western blot analysis on active phosphorylated form and total SMAD2/3 in total protein extracts of treated cells. Phospho-SMAD2/3 levels were corrected by total SMAD2/3 densitometry. Western blot data are presented as the fold change with respect to untreated target protein expression. The results are expressed as mean ± SEM (*n* = 3/group). One-way ANOVA and Bonferroni’s post-test: * *p* < 0.05. (**B**) Representative images of immunostaining for SMAD2 to visualize nuclear translocation. Nuclei are stained with Hoechst 33342. Magnification is 40× and the scale bar indicates 50 μm (*n* = 3/group).

To assess the molecular mechanism by which TGF-β1 activates the fibrotic process in ACM C-MSC, we performed a Western blot analysis on total protein extracts of C-MSC isolated from ACM and HC at early time points. We found significant activation of TGF-β1 canonical signaling pathway, in terms of higher expression levels of phosphorylated form of SMAD2/3 protein in TGF-β1-treated ACM C-MSC with respect to the untreated ones (Figure 6A: 1.000 ± 0.08636 a.u. in Untreated vs. 1.911 ± 0.3880 a.u. in TGF-β1 vs. 0.7580 ± 0.2064 a.u. in TGF-β1+LY, *n* = 3 each, *p =* 0.0317 and *p =* 0.0197 respectively). As expected, SMAD2/3 phosphorylation was down-modulated by LY. Since phospho-SMAD2/3 migration into the nucleus to activate pro-fibrotic transcription pathway is well known [21], we performed an immunofluorescence analysis to determine our in vitro context SMAD2 localization. Indeed, an enhanced SMAD2 nuclear localization was evident in the ACM group following TGF-β1 treatment (Figure 6B).

On the contrary, no significant differences upon TGF-β1 treatment were detectable for the phosphorylated forms of ERK1/2, the non-canonical TGF-β pathway, neither in HC nor in ACM C-MSC (Figure S1).

## 3. Discussion

In this work we provide, for the first time, evidence of the whole stromal cell compartment contribution in pathological fibrotic remodeling of an ACM heart. In particular, we demonstrated that the cardiac and systemic excess of TGF-β1 in ACM patients directly acts on ACM C-MSC, mediating the pro-fibrotic differentiation of these cells.

Nowadays, it is well known that the ACM heart commonly undergoes progressive replacement of the ventricular wall, by fibrotic or adipose tissue, leading to an impaired contractile function and constituting a non-conductive substrate, source of re-entrant arrhythmias.

Adipogenic remodeling is a well-studied process, mainly dependent on the non-contractile cardiac stromal compartment.

Myocardial fibrosis is a clinical feature that generally occurs following a cardiac injury. It starts as a reparative mechanism but can move to a persistent pathological status. ACM-specific fibrosis is an understudied phenomenon of excessive matrix and collagen deposition and follows the modification of mechanical/electrical properties, as described in other cardiac diseases [22].

The first result of our work, suggesting the potential role of C-MSC as myofibroblast precursors, concerns the presence of these cells (CD44 positive cells) in the highly fibrotic area of ACM biopsies. Indeed, mesenchymal stromal cells of different origins (from adipose tissue, umbilical cord, bone marrow or cardiac) can acquire a myofibroblast-like phenotype, which is characterized by α-SMA stress fiber expression and collagen production in standard cell culture conditions on plastic support [23]. All these features are enhanced by TGF-β1 treatment [23]. Unexpectedly, no difference was present in the expression of pro-fibrotic markers between C-MSC isolated from ACM RV endomyocardial biopsies and those isolated from HC samples in the standard culture condition. Nevertheless, TGF-β1 treatment interestingly unveiled a differential response of ACM vs. HC cells. Accordingly, we demonstrated, for the first time, increased levels of cardiac and circulating TGF-β1 in ACM patients in comparison with the HC subject, suggesting that the presence of a pro-fibrotic systemic environment can prompt cardiac fibrosis.

The source of excess TGF-β1 in ACM could speculatively be secondary to genetic causes or arise from environmental factors. As for the first hypothesis, it has been demonstrated that the TGF-β1/p38 MAPK pathway depends on *PKP2* and *DSP* expression. Loss of *Pkp2* in neonatal rat ventricular cardiomyocytes leads to the increase of both *Tgfb1* mRNA and TGF-β1 secreted in the supernatant as well as to increased gene expression of ECM components [24]. Moreover, *Pkp2* knockdown causes the loss of DSP expression. Since the restoration of DSP expression rescues the activation of TGF-β1/p38 signaling, DSP is thought to act upstream of TGF-β1/p38 and downstream of PKP2 [25]. Notably, it has been also reported that the upregulation of TGF-β1 is not only limited to *PKP2* haploinsufficiency, but also to the increased levels of plakoglobin observed in mutant mouse hearts [25]. In addition, the *Jup* deletion leads to the increase of Smad2 phosphorylation and TGF-β1 expression in mouse hearts and derived CM [24]. TGF-β expression changes can also occur upon direct genetic alterations. Different mutations have been identified in genes encoding non-desmosomal proteins [26] such as *TGFB3*, responsible for the ARVD1 form [27]. Mutations in *TGFB3* are linked to an increase in (i) cardiac fibrotic remodeling in ACM patients and (ii) in vitro *TGFB3* gene expression [27].

In addition to genetic predisposition, different triggers, typically occurring during ACM pathogenesis, can result in TGF-β1 production. Physical exercise is a recognized phenotypic modulator in ACM [28]. Athletes with a history of endurance sports exhibit increased levels of plasmatic TGF-β1 and develop myocardial fibrosis in contrast to novice athletes [29,30]. Intense physical exercise is both associated with activation of β adrenergic signaling and leads to excessive mechanical stretching of the heart. The areas affected by myocardial replacement in the ACM heart show reduced reuptake of norepinephrine that promotes stimulation of adrenergic receptors [31]. The norepinephrine treatment induces cardiac fibrosis and *TGFB1* gene expression in rats’ ventricular endocardium [32,33]. TGF-β1 overexpression, in turn, promotes an increased expression of β-adrenergic signaling [34,35,36]. In addition, TGF-β1 alters the electrophysiological crosstalk between myofibroblast and cardiomyocyte, resulting in a proarrhythmic phenotype [37]. Moreover, high-level sport activity implies excessive heart stimulation, causing the mechanical stretch of fibers, leading to TGF-β activation [38]. Indeed, TGF-β1 is secreted in an inactive form bound to a latent complex, consisting of latency-associated protein [19] and latent TGF-β binding protein 1 (LTBP-1). Mechanical stretching can induce the release of active TGF-β from the LTBP/LAP complex [38].

In addition, inflammation, which has been reported in 3/4 of ACM cases [39], may be a potential source of TGF-β1. In particular, chronic inflammatory processes, occurring during the progression of heart diseases, are responsible for the production of several cytokines, such as TGF-β [40]. In general, patients with heart failure are characterized by increased cardiac inflammation. Specifically, the inflammatory process is proven by the presence of CD3^+^/CD11a^+^/CD45^+^ inflammatory cells, which in turn are responsible for TGF-β1 secretion and for triggering cardiac collagen accumulation [41]. In response to an initial TGF-β trigger, we showed that ACM C-MSC are able to produce further TGF-β1 and collagen.

Our in vitro results show the key role played by C-MSC isolated from ACM patients, which exhibit the typical features of differentiated myofibroblasts as a result of the pro-fibrotic culture condition. This effect is limited to ACM-derived cells, while HC have a limited response to the TGF-β1 stimulus. Moreover, the rescue effect obtained by LY364947 treatment demonstrated that the effect is specifically due to TGF-β activity. It is well accepted that TGF-β directly mediates myofibroblast differentiation and ECM production through the activation of both canonical (SMAD2/3) and non-canonical (MAPK) signaling pathways, which in turn drive the regulation of fibrosis-mediating genes [12,42,43]. Our results further prove that the myofibroblast-like phenotype of ACM C-MSC depends on TGF-β-SMAD2/3 signaling activation.

We cannot exclude that other molecular mediators may participate in the establishment of fibrosis in ACM. In addition, different plasmatic markers of fibrosis were reported in ACM patients [2]. Specifically, circulating levels of the interleukin-33 receptor ST2, with a potential role in immune, pro-fibrotic response during myocardial injury, have been studied in ACM patients [44]. Similarly, differential concentration of galectin-3, which is involved in inflammation and fibrosis [20], were found in ACM vs. control plasma samples [45].

Our data expand and integrate what was already reported for fibro-adipogenic progenitors (FAP). This specific C-MSC population, isolated based on PDGFRα and Sca1 markers, was defined as bi-potential based on both fibrous commitment and fat gene expression. In human and mouse hearts, the FAP population has been implicated in ACM fibro-fatty substitution. Moreover, the deletion of DSP limited to cardiac FAP leads to an increased interstitial fibrosis with overexpression of TGF-β1 in mice ventricular myocardium [18,46].

## 4. Materials and Methods

### 4.1. Ethical Statement

This study complies with the declaration of Helsinki and was approved by the Centro Cardiologico Monzino Ethic Committee (R1020/19-CCM1072; date of approval: 3/7/2019). Written consent was signed by participating ACM patients (right ventricle endomyocardial bioptic samples and blood samples) and healthy control donors (blood samples). The healthy control (HC) right ventricle (RV) endomyocardial samples were obtained from cadaveric donors from the “Cardiovascular Tissue Bank” of Centro Cardiologico Monzino (MTA signed 5 November 2019). The supplementary Tables S2 and S3 summarize the clinical features of the enrolled ACM and HC subjects, respectively.

### 4.2. Plasmatic TGF-β1 Concentration Assay

For each enrolled subject (Table S1), 5 mL of peripheral whole blood was collected using EDTA Vacutainer tubes (Becton Dickinson, Franklin Lakes, NJ, USA). Plasma was obtained by centrifuging the whole blood at 1500 g for 15 min at 4 °C. Collected plasma samples were spun at max speed for 10 min at 4 °C and the supernatants were used for the assay. Hemolyzed samples were discarded. TGF-β1 plasma levels were detected by an ELISA kit (Human TGF-β1elisa kit, R&D Systems, Inc. Minneapolis, MN, USA) following the manufacturer’s instructions.

### 4.3. Immunofluorescence on Tissues and Cells

To perform the immunofluorescence assay, paraffin was removed from the embedded right ventricle (RV) endomyocardial bioptic sections from ACM patients and endomyocardial samples for HC donors. Antigen unmasking was performed by heating sections at 90 °C in antigen retrieval buffer pH 6 (DAKO, Santa Clara, CA, USA). For cell immunofluorescence, C-MSCs were fixed using 4% paraformaldehyde in PBS. After blocking with PBS supplemented with 5% BSA and 0.1% Triton X-100 (PBS-T/BSA) for 60 min, the slides were incubated with specific primary antibodies (as reported in Table S4) overnight (O/N) at 4 °C. As a negative control, species- and isotype-matched IgGs were incubated in the place of primary antibodies. Fluorescence-labeled secondary antibodies (Invitrogen, Carlsbad, CA, USA) were added for 1 h. Nuclei were stained with Hoechst 33342 (Sigma-Aldrich, Saint Louis, MO, USA). Images were acquired with a confocal microscope (Zeiss LSM710—ConfoCor3 LSM, Zeiss, Germany) using the software Zen 2008 (Zeiss, Germany).

### 4.4. C-MSC Isolation, Culture, and Treatment

Cells were obtained from patients’ endomyocardial biopsies, or from donors’ endomyocardial specimens, and characterized as previously described [47] and cultured with Iscove’s Modified Dulbecco’s Medium (Thermo Fisher Scientific, Waltham, MA, USA) supplemented with 20% Fetal Bovine Serum (FBS), 10 ng/mL basic fibroblast growth factor, 10,000 U/mL Penicillin, 10,000 µg/mL Streptomycin, and 0.02 M L-Glutamine. The treatments were performed after O/N growth in low serum medium (2% FBS) and specifically by adding 5 ng/mL of TGF-β1 (PeproTech, London, UK), supplemented or not with 10 µM of LY364947 (Sigma-Aldrich, Milan, Italy) for different times, specifically reported in each figure legend.

### 4.5. mRNA Extraction and qRT-PCR Assay

After collection, RV endomyocardial bioptic samples from HC donors and ACM patients were crushed by mechanical disruption using metallic-beads by a TissueLyser (Qiagen, Milan, Italy) in an appropriate amount of RL lysis buffer (Norgen Biotek corp., Thorold, Canada). Cell cultures were lysed in RL buffer. RNA, both from cells and tissues, was isolated by using a Total RNA Purification kit (Norgen Biotek corp., Thorold, Canada). The quantification of the isolated RNA was determined by a NanoDrop spectrophotometer (ND-1000, EuroClone, Milan, Italy). Reverse transcription was conducted with SuperScript III (Invitrogen, Carlsbad, CA, USA) following the manufacturer’s instructions. qRT-PCR was performed with the use of the iQTM SYBR Green Super Mix (Bio-Rad Laboratories, Hercules, CA, USA) and specific primers (reported in Table S5). All reactions were performed in a 96-well format with the 7900HT Fast Real-Time PCR System (Thermo Fisher Scientific, Massachusetts, USA). The relative quantities of specific mRNA were obtained with the use of the comparative Ct method and were normalized to the housekeeping gene glyceraldehyde 3-phosphate dehydrogenase (*GAPDH*).

### 4.6. Protein Extraction and Western Blot Analysis

Both RV samples and C-MSC were lysed in cell lysis buffer (Cell Signaling Technology, Danvers, MA, USA) supplemented with protease and phosphatase inhibitor cocktails (Sigma-Aldrich, Saint Louis, MO, USA). Biopsies were crushed by using metallic-beads in a TissueLyser (Qiagen, Milan, Italy). Total protein extracts were subjected to SDS-PAGE and transferred onto a nitrocellulose membrane (Bio-Rad, California, USA). The membranes were blocked for 1 h at room temperature in 5% non-fat dry milk in Wash Buffer (Tris Buffer Sulfate, 0.1% Tween-20) and then incubated O/N at 4 °C with the appropriate primary antibodies (reported in Table S4). The membranes were incubated with peroxidase-conjugated secondary antibodies (GE Healthcare, Chicago, IL, USA) for 1 h. Signals were visualized using the LiteUP Western Blot Chemiluminescent Substrate (EuroClone, Milan, Italy). Images were acquired with the ChemiDoc^TM^ MP Imaging System (Bio-Rad, California, USA), and densitometric analysis of membranes was performed using the ImageJ software (National Institutes of Health, Bethesda, MD, USA). Proteins from RV samples and C-MSC were normalized according to the Ponceau Red staining and glyceraldehyde 3-phosphate dehydrogenase (GAPDH) signal, respectively.

### 4.7. Statistical Analyses

Quantitative results are expressed as mean ± SEM. Statistical analysis was performed with GraphPad Prism 5. Quantitative variables were analyzed by one-way ANOVA with Bonferroni’s post-test or Student’s *t*-test, as appropriate. Categorical variables were compared with Fisher’s exact test. A value of *p* ≤ 0.05 was considered statistically significant.

## 5. Conclusions

In conclusion, we describe the role of C-MSC as a source of myofibroblasts in ACM hearts and highlight the consequence of the excess TGF-β1 as *primum movens* for ACM C-MSC pro-fibrotic commitment, happening through the SMAD2/3 pathway. Therefore, the whole process is the result of the influence of a pro-fibrotic microenvironment on genetically-predisposed C-MSC. These discoveries are relevant for ACM patient management, as we can foresee potential therapeutic interventions, by modulating the triggers of excessive release of TGF-β1. These could be achieved by restricting physical exercise and reducing altered inflammatory response.

## Data Availability

The data presented in this study are available on reasonable request from the corresponding author. The data are not publicly available due to privacy reasons.

## Supplementary Materials

The following are available online at https://www.mdpi.com/1422-0067/22/5/2673/s1, Figure S1: ACM C-MSC pro-fibrotic commitment does not involve ERK1/2 activation, Table S1: Demographic characteristics of subjects enrolled for plasma test, Table S2: Clinical data of ACM patients enrolled for biopsy samples, Table S3: Clinical features of the deceased tissue donors, Table S4: Primary antibodies, Table S5: Primer sequences 5′–3′.
