# Supplementary material for: Excess TGF-β1 Drives Cardiac Mesenchymal Stromal Cells to a Pro-Fibrotic Commitment in Arrhythmogenic Cardiomyopathy
Source: Int J Mol Sci. 2021 Mar 6;22(5):2673. doi: 10.3390/ijms22052673 (PMC7961797; doi:10.3390/ijms22052673)
Supplement: Supplementary file 1 [file ijms-22-02673-s001.pdf]

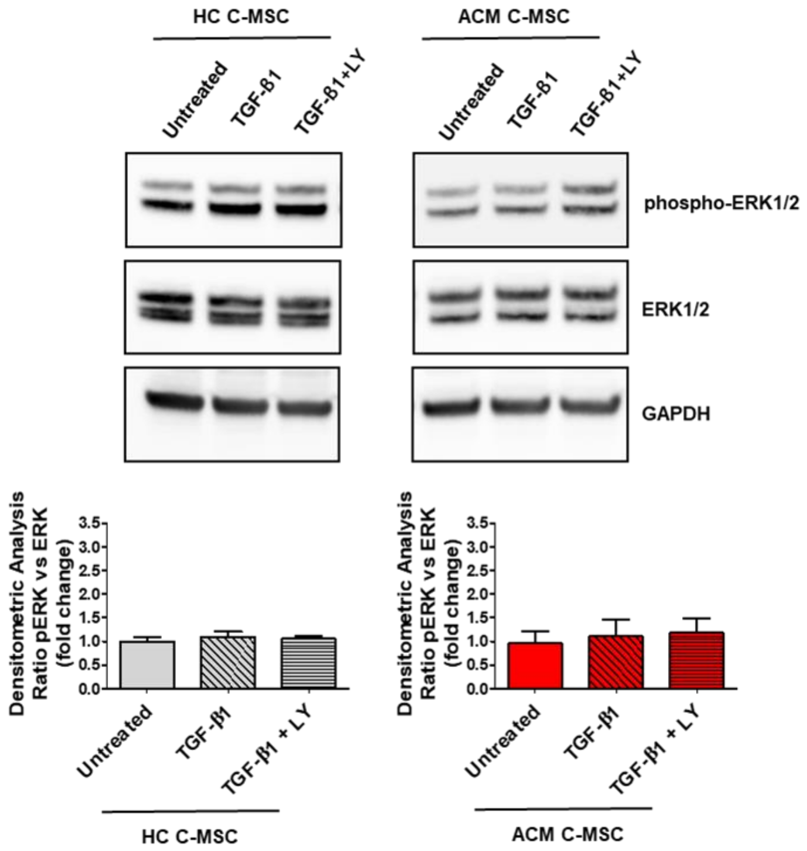

Figure S1. ACM C-MSC pro-fibrotic commitment does not involve ERK1/2 activation. Cardiac mesenchymal stromal cells isolated from HC donors and ACM patients were treated as described in Figure 6. Total protein extract from treated cells was subjected to Western blot analysis to visualize active phosphorylated form and total ERK using specific antibodies. Phospho-ERK1/2 levels were corrected by total ERK1/2 densitometry. Western blot data are presented as the fold change of target protein expression. The results are expressed as mean ± SEM, (n= 3/group). One-way ANOVA and Bonferroni's post-test: no significant difference.

Table S1. Demographic characteristics of subjects enrolled for plasma test

	ACM	HC	P value
Sex (% Male)	43/52 (82.69%)	45/52 (86.54%)	0.7866
Age (mean ± SE)	44.11 ± 1.94	42.09 ± 1.90	0.4583

Table S2. Clinical data of ACM patients enrolled for biopsy samples. Minor and major scores are given according to the International Task Force Criteria for the diagnosis of ACM [1] .VT: ventricular tachycardia; PVCs: premature ventricular contractions. Mutations are reported only when considered pathogenic or likely pathogenic.

	**Sex/Age (at recruitment)**	**Age/Type of first manifestation**	**Dysfunction/ structural alterations at imaging**	**Tissue characterization**	**Repolarization abnormalities**	**Depolarization conduction abnormalities**	**Arrhythmias**	**Family history/ Genetics**
ACM1	M/ 52	51/ VT	minor	minor	minor	minor	major	major (*PKP2* c.2013delC p.Lys672ArgfsX12)
ACM2	M/ 42	42/VT	major	not conclusive	major	no	major	major (*PKP2* c.1643delG p.Gly548ValfsX15)
ACM3	M/ 41	27/PVCs	major	not conclusive	major	no	minor	major (*PKP2* c.2013delC p.Lys672ArgfsX12)
ACM4	M/ 51	51/VT	minor	major	no	minor	major	no

ACM5	F/ 24	24/VT	major	not conclusive	minor	no	major	major (*DSG2* c.1003A>G p.Thr335Ala)
ACM6	M/ 50	41/ECG alterations	major	not conclusive	major	no	minor	no
ACM7	M/ 47	35/PVCs	minor	not conclusive	major	no	minor	no
ACM8	M/ 46	45/syncope	minor	not conclusive	minor	no	no	major
ACM9	M/ 43	43/VT	major	minor	minor	minor	major	no
ACM10	F/ 52	50/PVCs	major	major	minor	minor	minor	no

Table S3. Clinical features of the deceased tissue donors (with healthy heart) enrolled in this study.

ID	Sex (M=male; F=female)	Age	Cause of death	Concomitant diseases	Drugs	Cardiovascular risk factors
HC1	M	51	Cerebral hemorrhage	/	/	Hypertension
HC2	M	41	Multiple trauma	/	/	/
HC3	M	42	Multiple trauma	/	/	/
HC4	F	48	Cerebral hemorrhage	/	/	Hypertension
HC5	F	18	Multiple trauma	/	/	/

HC6	M	49	Cerebral hemorrhage	/	/	/
HC7	M	55	Cerebral hemorrhage	/	/	/
HC8	M	50	Multiple trauma	/	/	/
HC9	M	40	Cerebral hemorrhage	/	/	Smoking, Hypertension
HC10	F	50	Respiratory failure	Idiopathic pulmonary fibrosis	Angiotensin Receptor Blockers	/

Table S4. Primary antibodies

Protein	Clonality/Code	Source/Isotype	Company	Diluition
COL1A1	Monoclonal, #84336	Rabbit	Cell Signaling	WB: 1:1000; IF: 1:200
CD44	Monoclonal (Clone Hermes-1), ab119335	Rat	Abcam	IF: 1:50
α-SMA	Monoclonal, A 2547	Mouse IgG2a	Sigma-Aldrich	WB: 1:1000; IF: 1:200
TGF-β1	Monoclonal [9016], ab64715	Mouse IgG1	Abcam	WB: 1:1000

phospho-SMAD2/3	Monoclonal, D27F4	Rabbit IgG	Cell Signaling	WB: 1:1000
SMAD2/3	Monoclonal, D7G7	Rabbit IgG	Cell Signaling	WB: 1:1000; IF: 1:200
phospho-ERK1/2	Monoclonal, #4370	Rabbit IgG	Cell Signaling	WB: 1:1000
ERK1/2	Polyclonal, #9102	Rabbit	Cell Signaling	WB: 1:1000
GAPDH	Polyclonal (FL-335), sc-25778	Rabbit	Santa Cruz	WB 1:1000

Table S5. Primer sequences 5′ - 3′.

**Gene**	**Forward primer**	**Reverse primer**
COL1A1	CCCCTGGAAAGAATGGAGATG	TCCAAACCACTGAAACCTCTG
COL1A2	TCTAGAAAGAACCCAGCTCGCACA	TGCATCCTTGGTTAGGGTCAATCC
COL3A1	CCGCTAGAAACTGCAGAGACCTGAAA	ATCCTTGGTTAGGGTCAACCCAGT
ACTA2	TACTGCTGAGCGTGAGATTG	TTCTCAAGGGAGGATGAGGA

TGFB1	AAGTGGACATCAACGGGTTC	GTCCTTGCGGAAGTCAATGT
CTGF	ACCAATGACAACGCCTCC	TTGGAGATTTTGGGAGTACGG
GAPDH	ATGTTCGTCATGGGTGTGAA	GTCTTCTGGGTGGCAGTGAT

References

1. Marcus, F.I.; McKenna, W.J.; Sherrill, D.; Basso, C.; Bauce, B.; Bluemke, D.A.; Calkins, H.; Corrado, D.; Cox, M.G.; Daubert, J.P., et al. Diagnosis of arrhythmogenic right ventricular cardiomyopathy/dysplasia: proposed modification of the task force criteria. Circulation 2010, 121, 1533-1541, doi:10.1161/CIRCULATIONAHA.108.840827.
